# Non-Invasive Technologies in Wearable Glucose Monitoring: A Structured Overview for the Future

**DOI:** 10.3390/bios16080407

**Published:** 2026-07-26

**Authors:** Aqsa Imran, Muhammad Babar Ramzan, Laraib Hashmi, Sheheryar Mohsin Qureshi, Maham Raza, Shahood uz Zaman

**Affiliations:** 1 School of Engineering and Technology, National Textile University, Faisalabad 38000, Pakistan; draqsa@ntu.edu.pk (A.I.); laraib52hashmi@gmail.com (L.H.); rmaham232@gmail.com (M.R.); 2 School of Computing, Engineering and Physical Sciences, University of the West of Scotland, Paisley PA1 2BE, UK

**Keywords:** glucose, biosensor, non-invasive, invasive, minimally invasive, wearable monitoring

## Abstract

Diabetes management depends on regular glucose monitoring, yet conventional blood-based methods are invasive and can reduce user comfort. This review presents an overview of wearable glucose monitoring technologies, with emphasis on non-invasive approaches. It first distinguishes invasive, minimally invasive, and non-invasive monitoring and discusses the use of interstitial fluid, sweat, saliva, tears, urine, and breath as alternative sensing media. The review then summarizes optical, electrochemical, electrical/electromagnetic, and nanotechnology-enabled sensing methods, together with representative wearable and commercially reported devices. At the end, textile-based systems are compared with non-textile platforms in terms of comfort, flexibility, signal reliability, durability, and practical integration. Across these approaches, major limitations include variable relationships between alternative biofluids and blood glucose, interference from physiological and environmental factors, calibration requirements, motion artifacts, limited durability, and insufficient clinical validation. Future development requires more reliable sensing, improved wearable integration, standardized testing, and validation under real-world conditions.

## 1. Introduction

### 1.1. Diabetes

**Figure 1:**
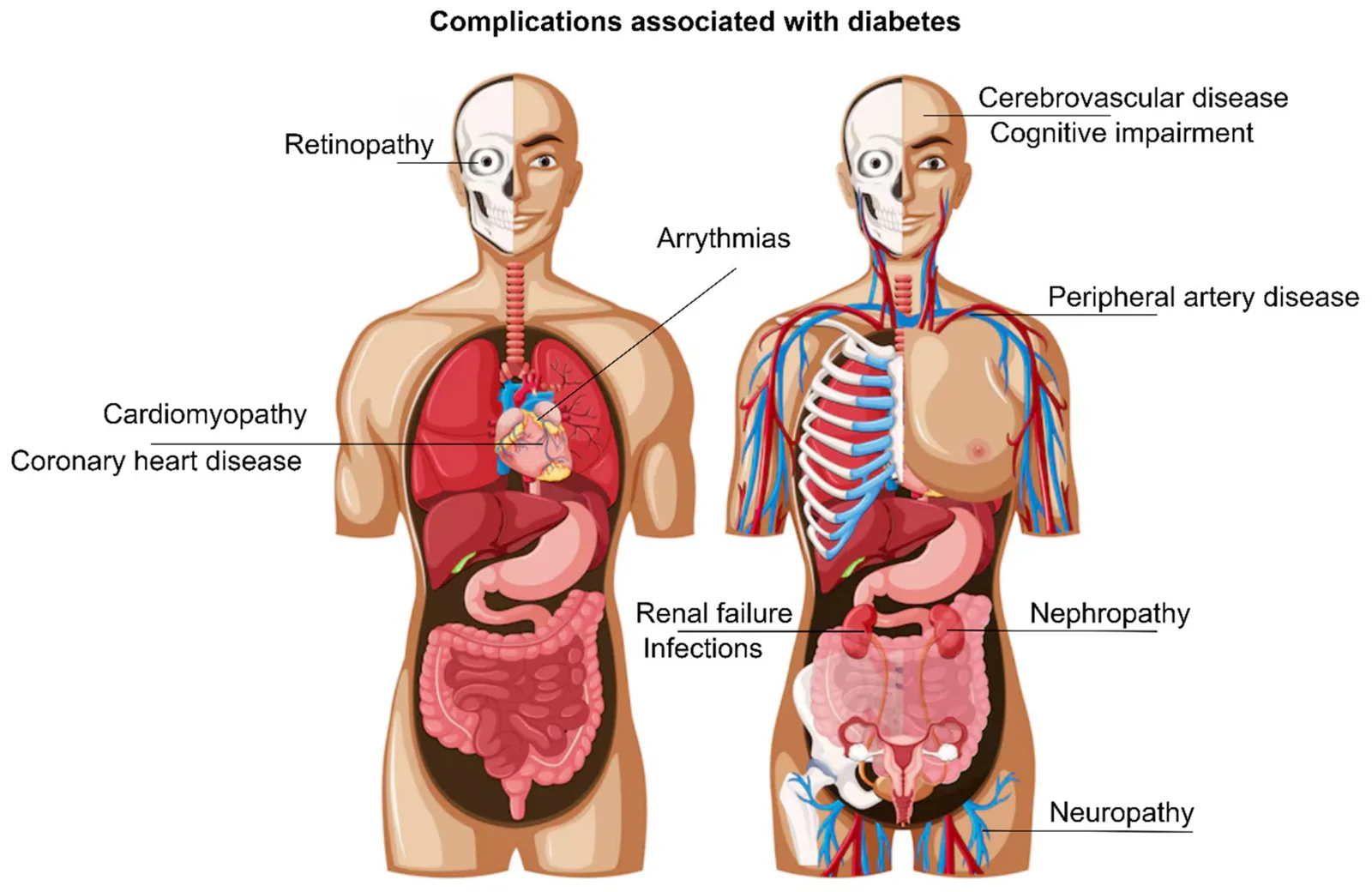
Complications associated with diabetes.

Diabetes mellitus (DM) or chronic hyperglycemia, mostly referred to as diabetes, is characterized by inappropriate levels of glucose in the blood [1]. This metabolic disease is slowly becoming a global threat since its occurrence is steadily increasing. According to the International Diabetes Federation (IDF), in 2013, the prevalence was estimated to be around 382 million [2], increasing to 415 million in 2015 [3], 425 million in 2017 [4], 463 million in 2019 [5], and 537 million in 2021 [6], and is predicted to reach above 700 million by the year 2045 [6,7]. Chronically raised levels of blood glucose become more life-threatening due to the complications associated with diabetes, including, but not limited to, growth disorders, diabetic ketoacidosis, hypoglycemia, endocrine disorders, elevations in problems associated with the liver, microvascular diseases and more [8]. The complications are compiled in Figure 1.

DM can be classified into several classes, some of which are only short-lived, such as gestational diabetes, while some affect children and can be due to genetic contributions [9,10]. Despite the various classifications, the major subsets of DM include type 1 (T1DM) and type 2 (T2DM), with the latter being the most common worldwide [11]. T1DM is due to inappropriate insulin action [12], while T2DM occurs due to inappropriate insulin secretion or insulin resistance, or a combination of both [11].

### 1.2. Treatment and Management

Insulin administration is one of the most widely known treatment options. The insulin may be administered via either continuous subcutaneous insulin infusion (CSII) using an insulin pump or by multiple daily insulin injections (MDI). The former can be connected to continuous monitoring techniques such as continuous glucose monitoring (CGM) sensors to ensure timely and effective medication administration and to help maintain the normality and quality of daily life [13,14,15,16,17]. For the management of diabetes mellitus, treatment/medication and monitoring go together. The disease needs to be managed by constant and consistent monitoring so that the treatment can be administered before worsening symptoms or the appearance of complications.

## 2. Methodology

**Figure 2:**
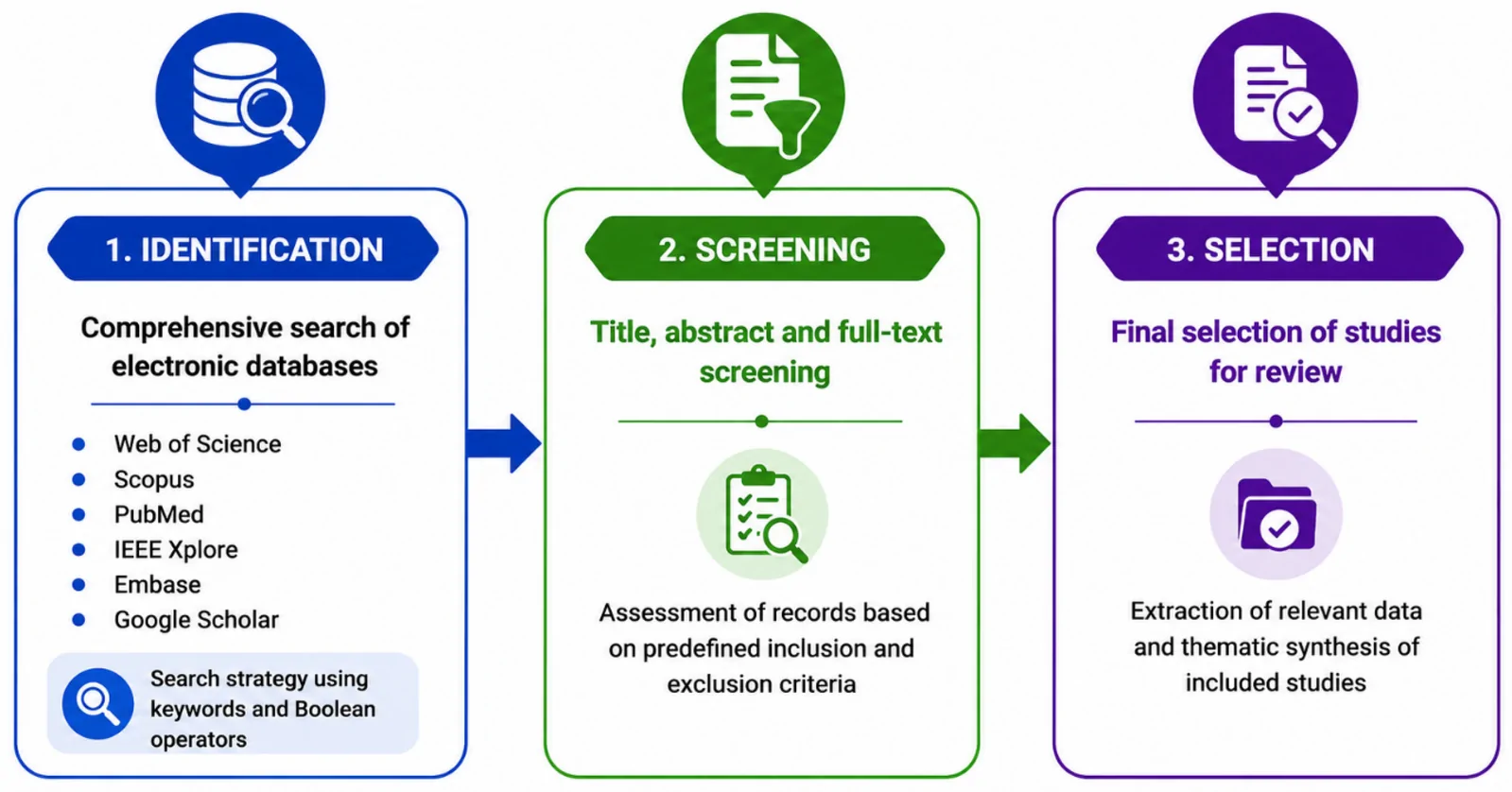
Methodology flow process.

In methodology, the selected literature provides a comprehensive dataset to analyze current technological advancements, integration strategies, and performance metrics for non-invasive wearable glucose sensors. The systematic extraction of experimental details, sensor configurations, and textile integration approaches enables a critical evaluation of each modality in terms of sensitivity, accuracy, and patient-centered design. This foundation allows the subsequent sections to present a detailed examination of monitoring methods, comparing invasive, minimally invasive, and non-invasive approaches while highlighting the specific opportunities and challenges associated with wearable systems. The following discussion synthesizes these insights to establish a clear technical overview for future research and clinical translation. The methodology flow process is shown in Figure 2.

### 2.1. Identification

A structured literature search was conducted across multiple scientific databases, including PubMed, Scopus, Web of Science, IEEE Xplore, Embase, and MDPI, to identify publications on non-invasive and minimally invasive wearable glucose monitoring. Keywords and Boolean operators such as “non-invasive glucose monitoring,” “wearable biosensor,” “electrochemical sensing,” “optical sensing,” “nanotechnology-enabled sensor,” and “continuous glucose monitoring” were employed to maximize search precision. The search covered studies published between 2018 and 2026. Following this identification phase, a total of 400 peer-reviewed articles were selected for further review, including both original research and review articles, ensuring comprehensive coverage of current technological developments.

### 2.2. Screening

The selected articles underwent a combined screening process, which included evaluation of titles, abstracts, and full texts. Studies were included if they focused on non-invasive or minimally invasive glucose sensing, reported quantitative performance metrics, and described methodological details such as sensor configuration, physiological fluid, and wearable integration. Exclusion criteria removed invasive-only systems, non-wearable prototypes, laboratory-only studies, or publications lacking validation data. This step ensured that only high-quality, relevant studies were retained for detailed technical analysis.

### 2.3. Selection

In the final selection phase, studies were systematically categorized into optical, electrochemical, nanotechnology-based, and electromagnetic/electric approaches. For each study, detailed data were extracted on sensor type, physiological fluid, and analytical performance. Comparative studies were constructed to summarize advantages, limitations, and translational potential. The data were further analyzed to identify technological bottlenecks, integration challenges, and opportunities for improvement, including the application of machine learning and data fusion for improved accuracy and signal stability. This structured methodology provides a rigorous framework for evaluating current glucose monitoring technologies across 325 cited references. A total of 75 references were excluded based on the above-explained exclusion criteria.

## 3. Monitoring Methods

**Figure 3:**
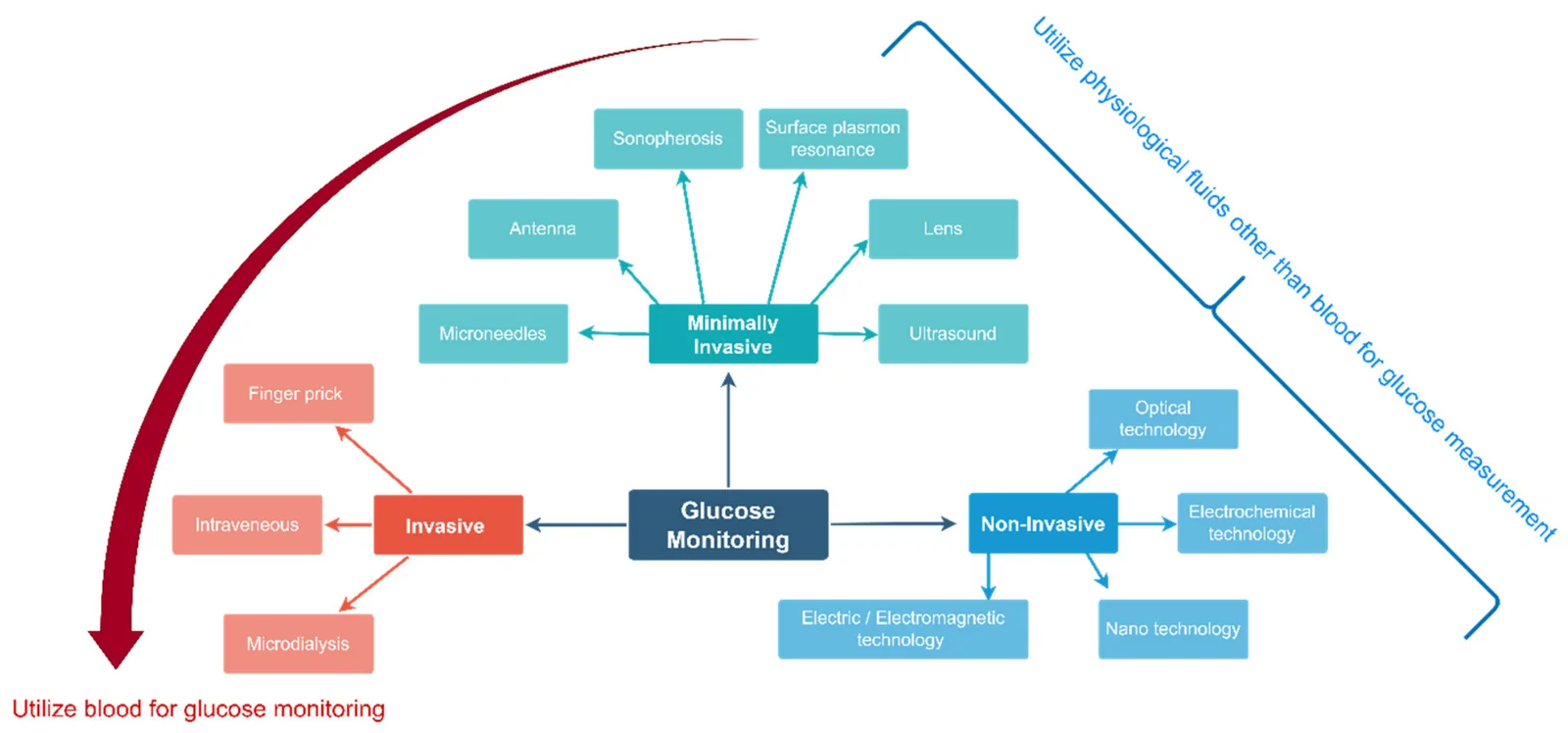
Glucose monitoring methods.

The monitoring of the disease for effective management depends on the determination of blood glucose levels and any fluctuations thereof. The traditional methods involve the use of a glucometer, micro-dialysis and/or laboratory testing, with the world slowly shifting towards continuous glucose monitoring (CGM) methods, all of which are invasive. Several advances are being made to develop, introduce, and commercialize minimally invasive and non-invasive methods to address problems involved with traditional invasive methods, such as the risk of infection, loss of sensation at the prick site, limited monitoring, inconvenience, cost limitations, repeated foreign body insertion, medical as well as general waste generation and more. The methods can be classified broadly based on whether they provide consistent and continuous monitoring as well as the mode of extraction of glucose. The former can be classified into continuous and intermittent monitoring, while the latter can be classified according to the degree of invasiveness. An overview of the available monitoring methods based on the level of invasiveness is compiled in Figure 3.

### 3.1. Invasive Methods

Traditional glucose monitoring involves blood sampling using glucometers, laboratory-based biochemical assays, or micro-dialysis. Electrochemical glucose monitoring primarily relies on enzymatic reactions, with glucose oxidase (GOx) and glucose dehydrogenase (GDH) being the most commonly used enzymes. In GOx-based systems, glucose is oxidized to glucono-δ-lactone, generating hydrogen peroxide, which is subsequently detected electrochemically to determine the glucose concentration. In GDH-based systems, glucose oxidation is coupled with electron transfer through cofactors such as nicotinamide adenine dinucleotide (NAD^+^) or flavin adenine dinucleotide (FAD), producing an electrical signal proportional to the glucose concentration. In contrast, laboratory-based glucose analysis commonly employs the hexokinase method, in which glucose is first converted to glucose-6-phosphate by hexokinase and subsequently oxidized by glucose-6-phosphate dehydrogenase to produce 6-phosphogluconate and NAD(P)H. The amount of NAD(P)H formed is proportional to the glucose concentration and is measured spectrophotometrically [18,19,20,21,22,23].

Self-monitoring of blood glucose or SMBG via glucometer has emerged as the most important tool for the effective management of diabetes. Glucometers are essentially handheld devices with a reading port onto which a calibrated strip with a blood drop received by a finger prick is installed [21,24,25,26]. The micro-dialysis technique involves inserting the probe into the abdominal wall and also utilizes a real-time monitoring unit via which continuous subcutaneous tissue glucose concentration can be measured precisely [27,28,29].

The major challenges associated with the traditional methods are the invasive nature and intermittent monitoring. This leads to problems such as inconvenience, infection and irritation at the prick site, an unsustainable nature since test strips cannot be reused, the requirement for a caregiver, possible injuries and mishandling, and more. To combat this, minimally invasive as well as non-invasive methods have been developed.

Commercial devices like Abbott FreeStyle Freedom Lite by Abbott Laboratories, Illinois 60064-3500United States, AgaMatrix WaveSense KeyNote by AgaMatrix, Inc., Derry, NH 03038, USA, Arkray Glucocard X-meter by ARKRAY USA, Inc., Minneapolis, MN 55402, USA, Bayer Ascensia Contour by Ascensia Diabetes Care, Basel, Switzerland, Bionime Rightest GM-300, Bionime Corporation, Taichung City, Taiwan, Nova Max, byNova Biomedical Corporation, Waltham, MA 02453-3432, USA, Roche Accu-Chek Aviva, by Roche Diabetes Care, Inc., Mannheim, Germany, and Advocate Redi-Code, by Pharma Supply, Inc., Wellington, FL 33414, USA, demonstrate rapid, validated glucose detection using enzymatic and electrochemical approaches shown in Table 1.

### 3.2. Minimally Invasive Methods

**Figure 4:**
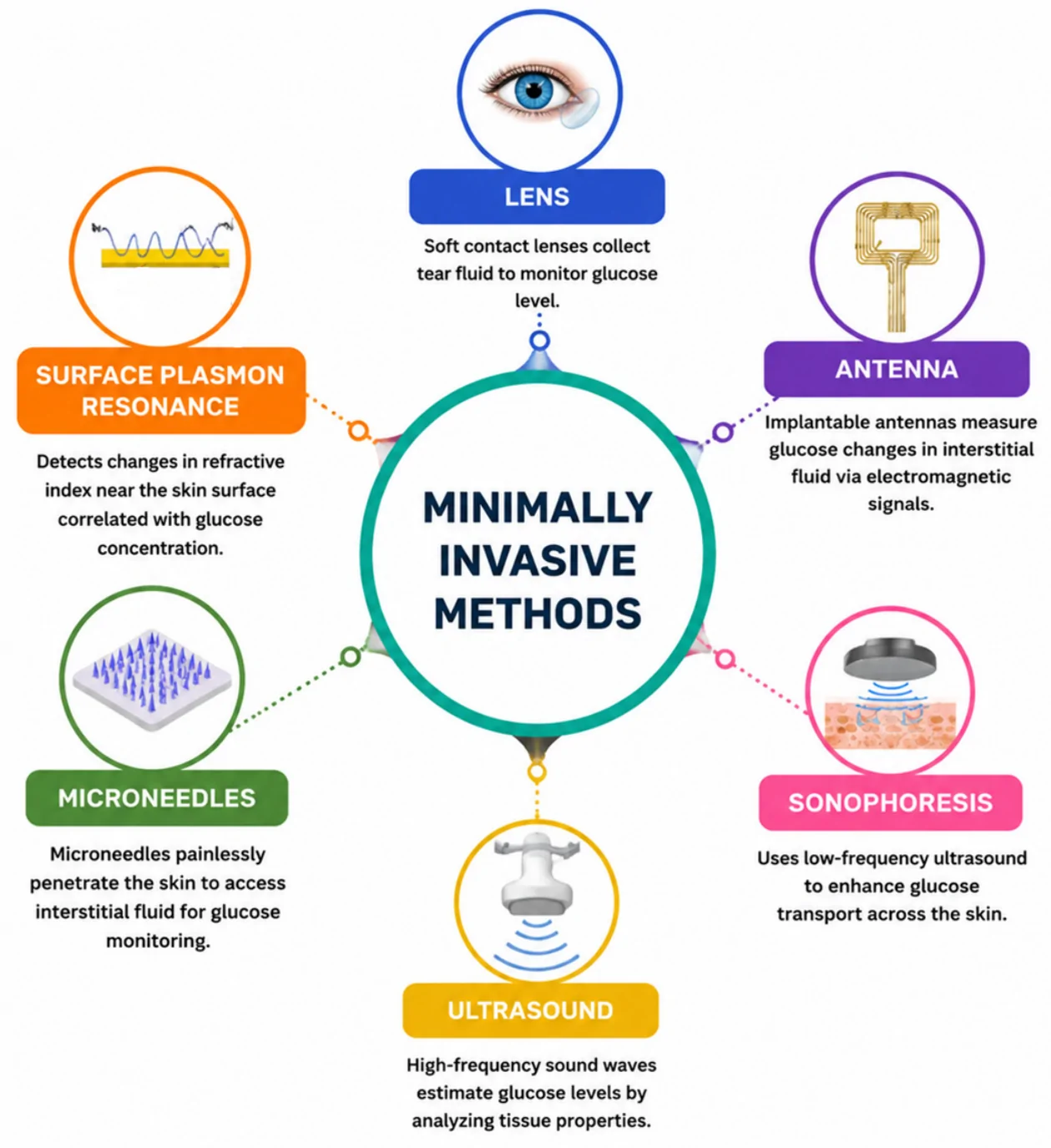
Minimally invasive techniques for measuring blood glucose.

Minimally invasive methods, at the core, are similar to invasive methods but utilize some other physiological fluid for glucose detection and are engineered specifically to be pain-free, efficient, and effective monitors of blood glucose levels. They are called minimally invasive since some form of bodily fluid, such as tears, interstitial fluid, etc., needs to be extracted to obtain a glucose sample. This involves an outside object being inserted into the body to obtain a sufficient sample that will provide a glucose reading. The main objective of this type of glucose monitoring method is to reduce the number of pricks or the interaction of bodily fluids with foreign objects and to provide continuous monitoring [18,25,31,32,33,34,35]. A compilation of the techniques utilized in this regard is given in Figure 4.

The biomarkers used are interstitial fluid and tears. Interstitial fluid contains similar levels of biological analytes, biomarkers, and molecules as those present in the blood and body. This causes it to be one of the most effective, accurate, and significant ways for determining body molecules such as glucose [25,32,33,35,36]. Extraction of interstitial fluid is achieved via microneedles and pain-free lancets. Different application modes can be applied.

Many tools and techniques that involve minimal invasivity to the patient and ensure continuous monitoring have been researched, developed, and are currently being utilized, as shown in Table 2. This includes the use of equipment such as lenses [37,38], antennas [39,40,41] and microneedles [32,42,43,44,45,46] or the utilization of techniques such as surface plasmon resonance [24,34,47,48], ultrasound [42] and sonophoresis [38].

### 3.3. Non-Invasive Methods

The most significant challenge for the monitoring of any medical discomfort or abnormality, especially for a disease whose complications and future implications can be severe and potentially painfully fatal, is consistent monitoring as well as efficiency. Comfort, both psychological, physical, and physiological, is necessary as well, as shown in Figure 3. All these factors, if met, produce a patient-centric approach. This can be achieved using non-invasive mechanisms.

### 3.4. Non-Invasive Monitoring

Non-invasive methods are patient-focused approaches prioritizing patients’ health on all possible fronts. Multiple wearables have been developed, and several advances are being made in this regard. In this regard, the major problem is the continuous extraction of body glucose samples.

#### 3.4.1. Physiological Fluids Involved

Since the extraction of blood is invasive, no matter how small the pain or prick is, other physiological fluids are to be utilized for the determination of blood glucose levels. These fluids need to provide a compatible level of glucose and need to always be readily available for extraction. The extraction should not rely on the insertion of a foreign object into the body. Sweat contains glucose in micromolar to millimolar levels and shows moderate correlation, but is affected by variable secretion, evaporation, and pH. Saliva has lower glucose concentrations and weaker correlation, influenced by food, oral health, and dilution. Tear fluid is suitable for ocular sensors but is limited by small sample volume and secretion variability. Urine glucose indicates hyperglycemia indirectly and is unsuitable for real-time monitoring. Breath acetone reflects metabolic changes linked to glucose but is strongly influenced by diet, metabolism, and environment. Each fluid offers potential for non-invasive sensing but presents practical challenges for consistent and accurate measurement as presented in Table 3.

##### Microfluidic Biofluid Sampling and Handling Systems

Microfluidic technologies have emerged as a critical enabling component in wearable non-invasive glucose monitoring systems by facilitating the controlled collection, transport, storage, and analysis of physiological fluids. While considerable research has focused on improving sensor sensitivity and selectivity, the reliability of glucose measurements is equally dependent on efficient biofluid management and sample delivery to the sensing region [59,60,61,62].

In sweat-based monitoring platforms, microfluidic networks composed of microscale channels, reservoirs, and sensing chambers enable continuous collection and transport of newly secreted sweat while minimizing contamination, evaporation, and sample mixing. Passive capillary-driven transport mechanisms are frequently employed to eliminate the need for external pumping systems, thereby enhancing wearability and reducing power consumption [59,60]. Epidermal microfluidic devices have demonstrated the ability to continuously monitor sweat biomarkers while maintaining user comfort and sample integrity during prolonged wear [59].

Recent wearable microfluidic systems have integrated electrochemical and colorimetric glucose sensors directly within microchannel architectures, allowing real-time biochemical analysis and improved measurement consistency [61,62]. Furthermore, multiplexed microfluidic platforms enable simultaneous monitoring of glucose alongside pH, temperature, electrolytes, and hydration markers, which can be utilized to improve calibration models and compensate for physiological variations (Table 4) [63,64].

Microfluidic technologies have also been investigated for saliva and tear-fluid monitoring. In saliva-based sensing systems, microchannels facilitate controlled transport of low-volume samples while minimizing contamination from food residues and oral debris [65]. Similarly, contact lens-based biosensors employ microfluidic structures to regulate tear collection and enhance interactions between tear fluid and sensing elements [66].

Despite these advantages, several challenges remain, including biofouling, channel blockage, evaporation losses, variability in biofluid secretion rates, and long-term mechanical durability. Future research should focus on antifouling materials, self-cleaning microfluidic architectures, stretchable substrates, and integration with machine learning-assisted calibration approaches to improve the reliability and clinical applicability of wearable glucose monitoring systems [63,64].

After the extraction of physiological fluid, the glucose level needs to be sensed and then converted into a comprehensible form. This can be done with various tools and techniques. The correlation between non-invasive detection of glucose in alternative biofluids and blood glucose is highly variable and fluid dependent. Sweat glucose demonstrates moderate correlation (R^2^ = 0.7–0.85) [67], but readings are affected by sweat rate, hydration status, pH, and local contamination. Saliva and tear glucose present weaker correlations (R^2^ = 0.5–0.8) [68] due to flow variability and limited volume. Breath-based volatile markers such as acetone show indirect correlation (R^2^ = 0.5–0.6) and are strongly influenced by diet, metabolism, and temperature [69]. These physiological interferences highlight the necessity of signal calibration, multi-modal data fusion, and algorithmic correction to enhance reliability.

#### 3.4.2. Methods of Non-Invasive Glucose Monitoring

**Figure 5:**
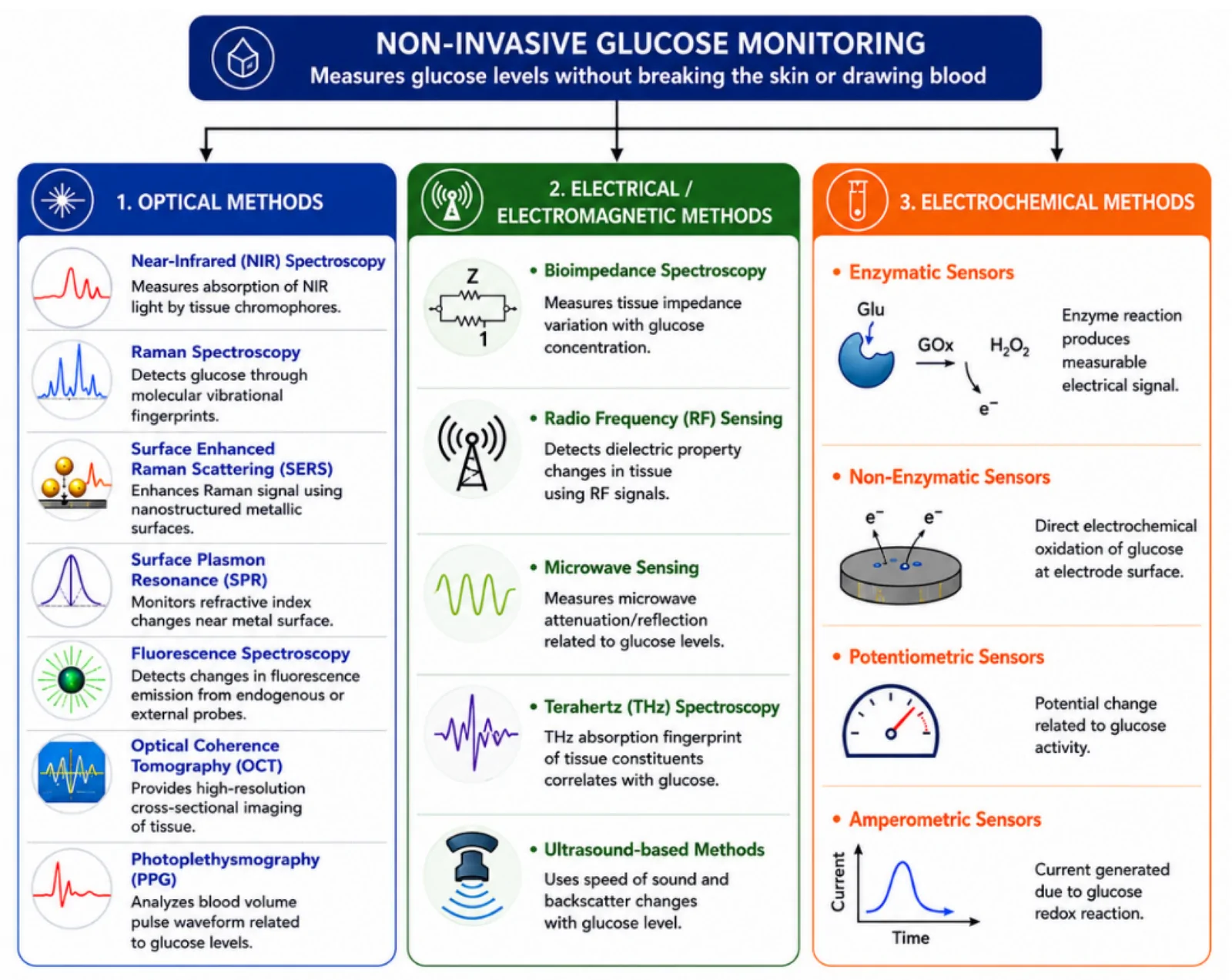
Non-invasive techniques for glucose monitoring.

As of 2026, various methods have been researched and are being utilized in the field of non-invasive glucose monitoring. Optical methods use the changes brought about by the interaction of light and glucose molecules in the physiological fluids explained above. Nanotechnology involves the use of nanomaterials for glucose sensing. Electrochemical methods for development of non-invasive sensors are similar to electrochemical methods as provided in the minimally invasive and invasive techniques with the only difference being changes in the glucose sample extraction and/or physiological fluid employed. Electromagnetic methods utilize the dielectric properties of glucose and sense the molecules in physiological fluids other than blood to produce comprehensible reading. All these methods can be continuous or intermittent. Oftentimes a combination of 2–3 methods/techniques is required to develop a reliable glucose monitoring smart wearable or E-textile (Electronic textile), since they enhance cross-validation of data (Figure 5).

**Figure 6:**
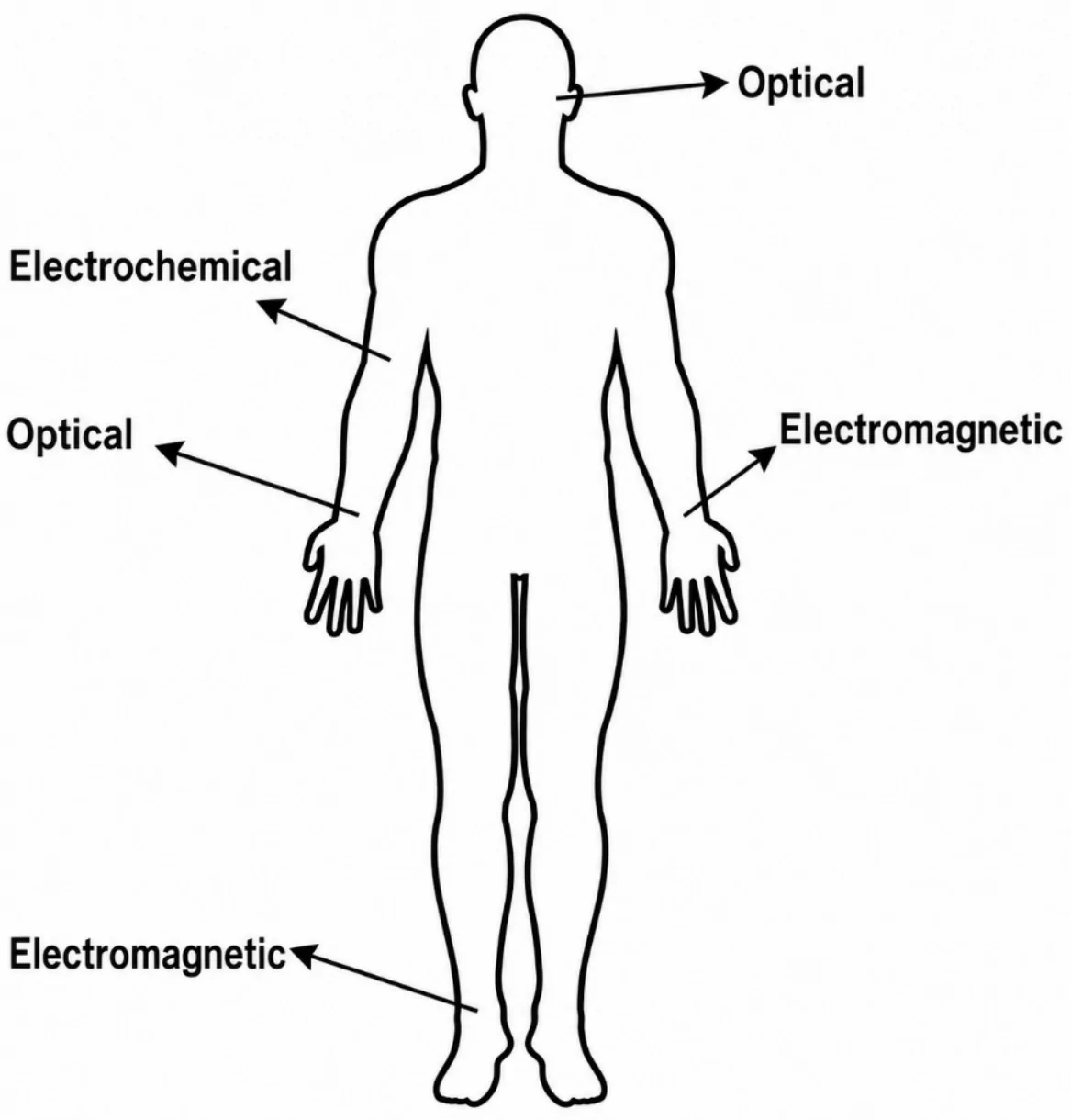
Continuous glucose sensing sites.

The development of multi-modal e-textile wearable can potentially revolutionize diabetes management by providing an accurate and holistic approach towards monitoring the disease. Depending upon the type of technique/s being used, the site of extraction differs. For continuous glucose monitoring via non-invasive methods, the extraction site of the physiological fluid needs to be at a place that does not cause problems or inconvenience in daily life and regular activities provide a regular and continuous level of the physiological fluid for which it is developed and easier to access for the patient [70,71]. A diagram in this regard is given in Figure 6.

Several continuous glucose monitoring devices have been produced by prominent medical and healthcare organizations such as Abbott, AgaMatrix, etc., and are now commercially available and in use by patients [31,37,71].

#### 3.4.3. Optical Methods

Various optical technologies have been modified and developed to be utilized for non-invasive monitoring of blood glucose. General physiological sites for measurement of glucose via optical techniques include the ocular humor and fingertip for intermittent monitoring and pulse areas such as the arm or the wrist for continuous monitoring. The utilization of optical fibers for glucose measurement depends on the linear relationship between the reflective index (RI) and glucose concentration [72] and/or the interaction between light of various wavelengths and glucose concentration.

**Figure 7:**
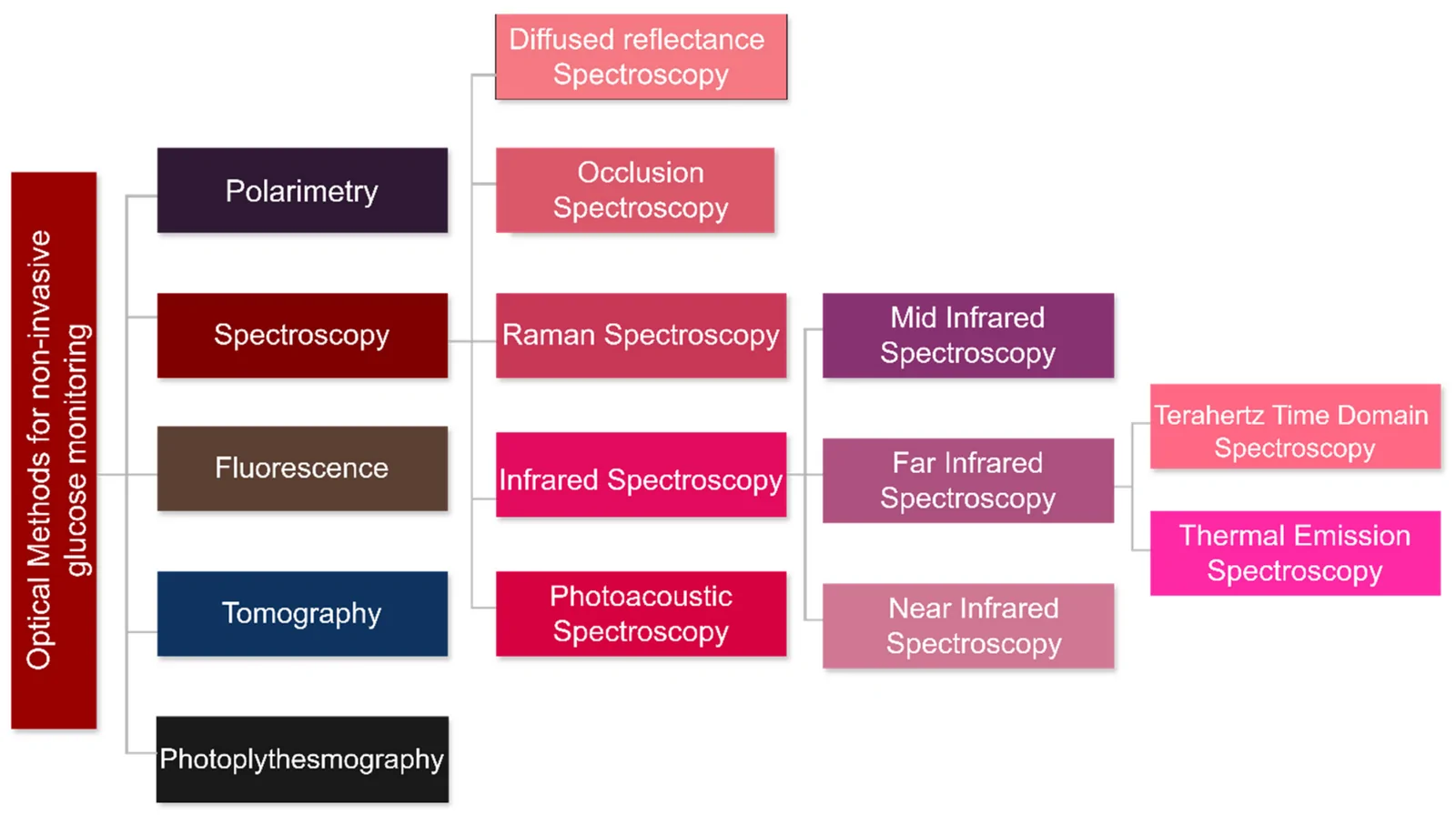
Optical methods used for non-invasive glucose monitoring.

Changes in reflective index as a result of changes in glucose concentration can either be due to the oxidation reaction of glucose, during which the transfer of flavin adenine dinucleotide (FAD) causes changes in the RI which can be picked up by the optical sensor and turned into a comprehensible reading, or due to covalent binding of glucose with other molecules such as concanavalin A (con A), glucose-binding protein (GBP), etc., which causes changes in RI significant enough to be tracked [73]. Several optical principles can be used for measurement as given in Figure 7.

Optical coherence tomography (OCT) uses light interference to analyze changes in tissue structure, considering the optical rotation angle of glucose. The fingertip can be used as a sensing site as developed by Chen T. et al. [74] and progress was made in accuracy enhancement by Miura et al. [75]. Optical polarimetry (OP) uses analysis of rotation and absorption of light by the glucose molecules, particularly in the aqueous humor. A non-contact glucometer consisting of a light source, photodetectors, beam splitters, and a processing unit utilizing the concept of OP for glucose detection has been developed and tested using serum glucose levels of rabbits by Hwang et al. [76] while a novel and low-cost detection system boosted using machine learning techniques has been produced by Bai et al. [77]. Furthermore, Stark and Arrieta have proposed a polarimeter setup with a Partial Least Squares regression algorithm to differentiate between glucose and albumin [78].

Photoplethysmography (PPG) analyzes blood volume changes due to infrared light directed at the site. The PPG sensors have been classified by the sensing site they utilize for the detection of glucose levels including wristband type, forehead type, and ear type [79,80]. Sensors utilizing the PPG principle have been developed for the early detection of diabetes with some sensors integrating machine learning for algorithm development and validation [81].

The near-infrared spectroscopy utilizes glucose’s ability to absorb light at specific wavelengths, so light is emitted in the range of 750 nm to 2.5 µm onto the skin. The reflectance is then analyzed [82,83]. Significant progress has been made in this regard by Han T. et al. [84] and Heise H. et al. [85] as well as Han G. and Chen [86]. The latter authors have proposed neural networks for better performance. Integration of machine learning for enhancement of results is the most prevalent in this type of optical sensor [82]. The mid-infra-red spectroscopy technique is characterized by a distinct absorption spectrum of glucose and so light is emitted in the range of 2.5 to 25 µm which interacts with the glucose molecules present in the interstitial fluid. Optical lasers are utilized in this regard and generally, a combination of two or more techniques is used. An efficient sensor has been developed by Mandal & Manasreh [87]. Lubinski et al. utilized mid-infrared technology for photothermal detection [88]. A combination of mid and near-infrared spectroscopy may also be utilized to analyze the absorption of glucose in blood. Machine learning is preferred for appropriate analysis [89]. Far infrared spectroscopy analyzes vibrational and rotational properties of glucose in the emitted light range of 0.3 to 30 THz [90,91].

A more recent and enhanced approach to far infra-red spectroscopy is Terahertz Time Domain spectroscopy which includes measurement of time delays in reflection for a more enhanced analysis [92,93,94]. Significant progress has also been made in occlusion/scattering spectroscopy which operates on the concept that the higher the glucose levels, the greater the scattering of emitted light, and has provided promising results for the future. One such device whose efficacy has also been tested is OrSense’s NBM device [90,95,96].

Another optical spectroscopy method being employed and tested for non-invasive glucose monitoring is the photoacoustic method. As glucose absorbs light in the infrared region, it produces vibration and sound, and the acoustics of that sound are analyzed. Single waves as well as differential waves have been used so far in the development of this method [90,97,98,99]. Diffusion reflectance spectroscopy utilizes near-infrared light to analyze tissue’s absorbance co-efficient parameters [100,101].

Fluorescence has also been proven to be a great method for non-invasive glucose measurement, especially from a miniaturization and smart wearable sensing perspective. Plasmon and carbon quantum dots have been utilized for this purpose so far. The plasmon-enhanced fluorescence is dependent on the interaction between plasmonic nanoparticles and glucose electromagnetic field which causes changes in fluorescence intensity. Similarly, carbon quantum dot fluorescence analyses fluorescence emissions via the implementation of carbon quantum dots obtained by the fusion of carbon nanoparticles [102,103,104,105,106,107,108,109,110].

Surface plasmon resonance utilizes electromagnetic field interaction with metallic nanoparticles in the presence of emission of visible light at the site. This causes changes in the refractive index via which shifts in resonance peaks occur. These shifts are then used as markers for determination of glucose levels [111,112,113,114].

Raman spectroscopy is also recognized as a promising method for non-invasive glucose measurement and has been improved upon by surface-enhanced Raman spectroscopy (SERS). The direct detection of glucose via SERS is difficult since glucose has a low cross-section and has poor adsorption on base metals. Several methods have been proposed so far to address these challenges. The four general categories of glucose sensing via SERS include active platform, partition layer functionalized surface, boronic acid-based sensors, and enzymatic reaction-based biosensors [102,111,115,116,117,118].

#### 3.4.4. Electrochemical Methods

**Figure 8:**
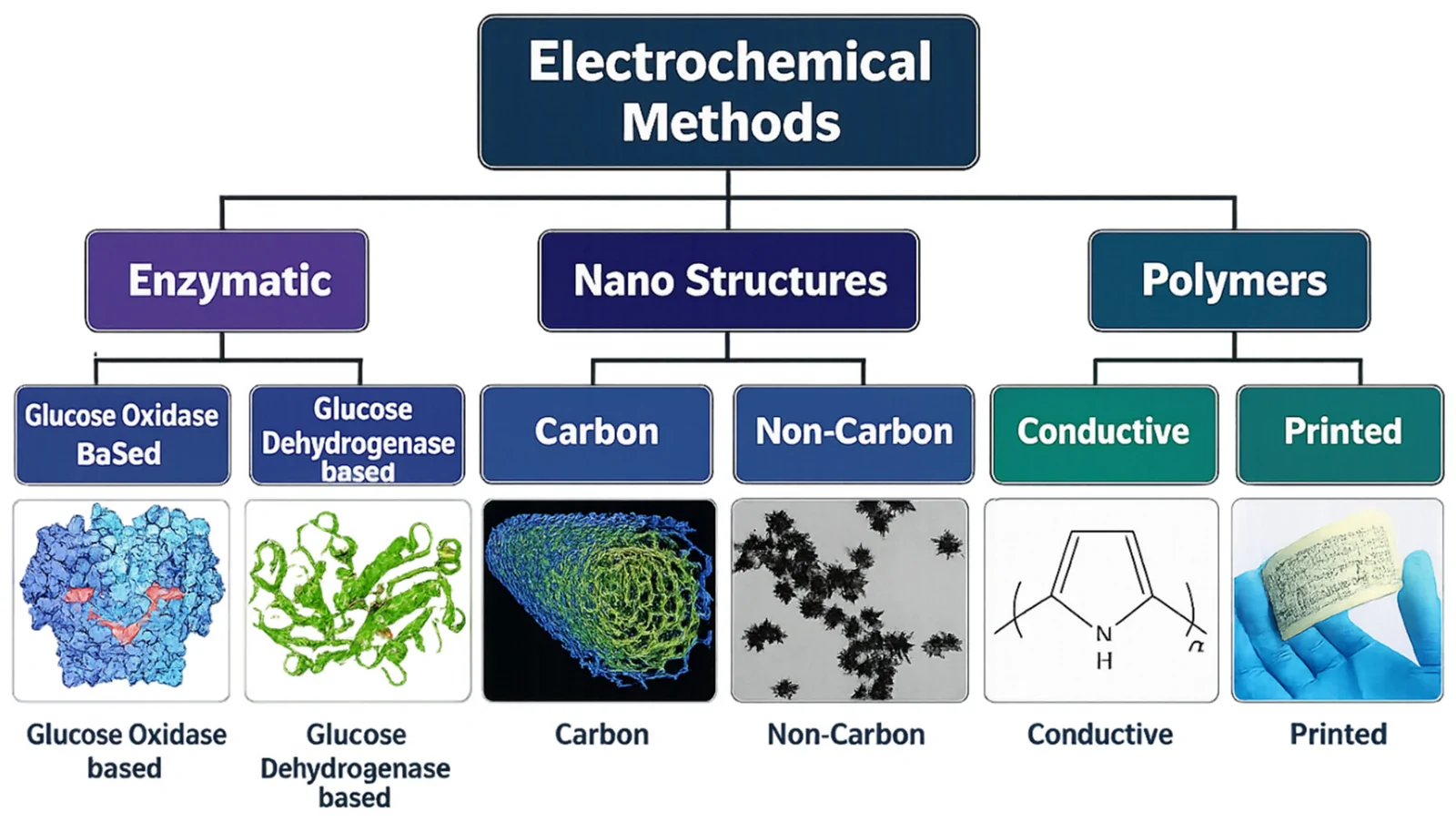
Electrochemical methods for non-invasive monitoring of glucose.

The electrochemical method utilizes enzymatic or non-enzymatic materials to detect oxidation and/or reduction of glucose molecules. The main objective is to analyze the chemical reaction that occurs due to the interaction between glucose and the enzyme/other chemical species. A compilation of techniques in this regard is given in Figure 8, which summarizes the main electrochemical sensing categories used for non-invasive glucose monitoring. The classification includes enzymatic approaches based on glucose oxidase and glucose dehydrogenase, nanostructure-assisted sensing systems using carbon and non-carbon materials, and polymer-based platforms including conductive and printed electrodes. This categorization helps to link the electrochemical sensing mechanism with the major material and fabrication strategies discussed in this section.

The non-enzymatic methods utilize nanostructures of carbon, cobalt, copper, nickel, and noble metal. The method is based on the interaction between glucose and these species. The oxidation reactions are then measured and analyzed for a comprehensible reading generation (see Section 3.4.6). Other works done in this regard include the utilization of an ‘‘incipient hydrous oxide adatom mediator’’ to analyze the glucose’s electrocatalytic process [119] and the utilization of metal-based catalysts in the electrode [120,121,122,123]. Electro-spun nanofibrous membranes also hold promising results since they can detect low concentrations of glucose with high sensitivity [124]. Another approach is the use of printed or conductive polymers. This approach also requires either an enzyme for functioning or carbon-based/non-carbon-based electrodes for glucose measurement [125,126].

**Figure 9:**
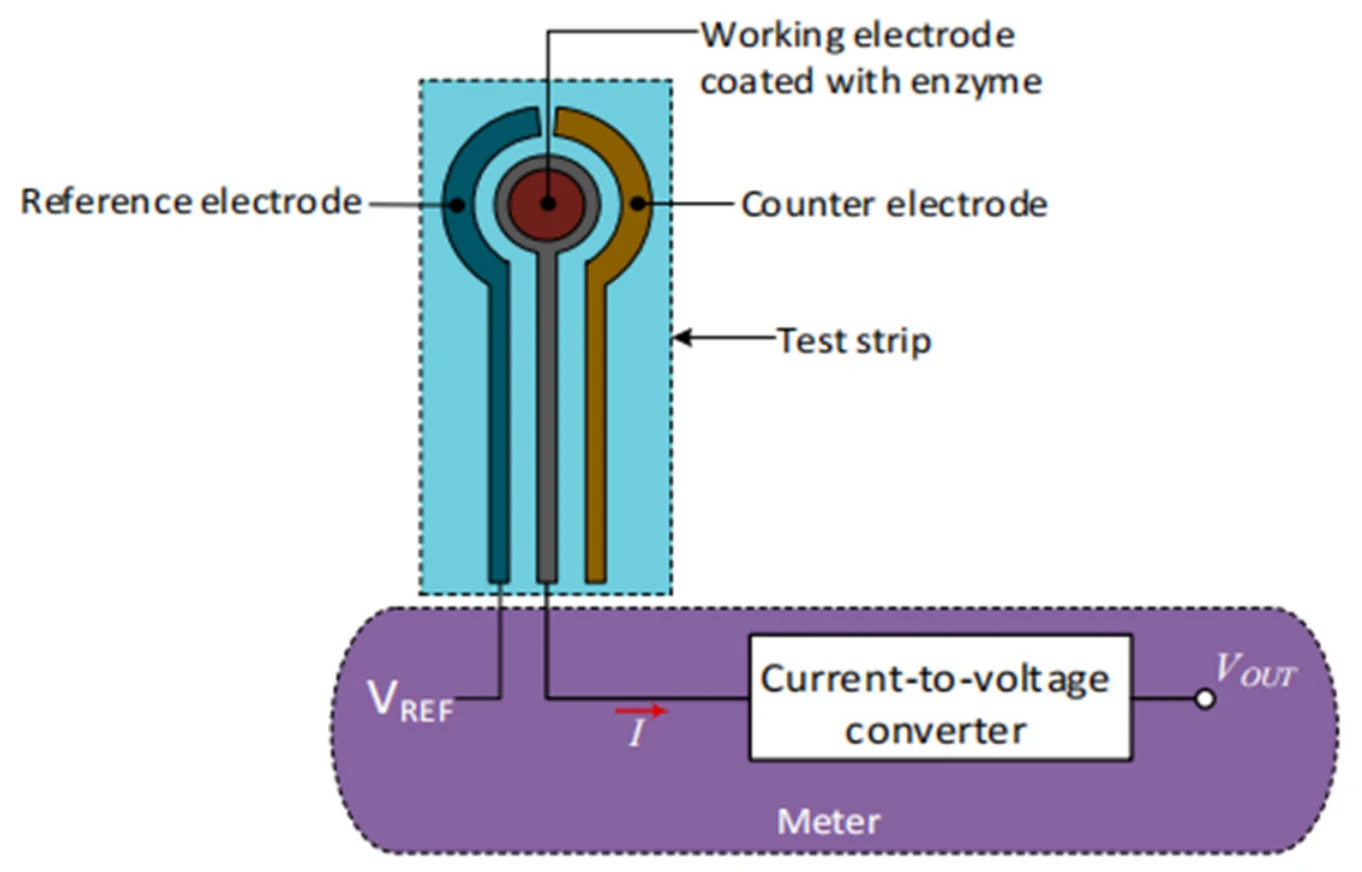
Block diagram of an enzyme-based glucose monitor.

The enzymatic methods utilize either GDH or glucose oxidase enzymes, the former of which is also heavily used in glucometer test strips. Immobilization of glucose oxidase on platinum electrode produced the first ever enzymatic electrochemical sensor. Enzymatic electrochemical sensing is still heavily researched from the perspective of glucose oxidase only, mainly because of the higher number of complications that need to be dealt with when using GDH and its heavy dependence on cofactors for proper functioning [127,128,129,130,131,132,133,134]. The working electrode consists of an enzyme, typically glucose oxidase or glucose dehydrogenase (GDH) attached to a coenzyme such as pyrroloquinoline-quinone PQQ or flavin adenine dinucleotide FAD and senses the chemical reaction. The counter electrode provides current to the working electrode and the reference electrode holds the voltage to aid with the reaction. The glucose oxidation in the presence of the enzyme produces current which is converted into voltage by the meter. The voltage value corresponding to the glucose concentration is displayed on the screen and read [18,25,26,135]. A block diagram is given in Figure 9.

The first generation of enzyme-based glucose sensors relied on the production of hydrogen peroxide (H_2_O_2_) during enzymatic reactions. The major issues involved were the requirement for high operating potentials, significant interference, as well as the fact that oxygen was required as a mediator. To resolve these issues, membranes such as Nafion, polypyrrole, polycarbonate, etc., along with the use of metalized carbon, Prussian blue, and metal hexacyanoferrate transducers are utilized [136,137,138,139,140,141,142,143,144,145,146,147,148,149]. Therefore, work in this regard includes sensors developed by incorporating rhodium particles within a nafion film, developing a glucose oxidase and tin oxide nanofiber film on a gold electrode modified with Prussian blue, integration of glucose oxidase with a catalytic polymer or the use of platinum nanoparticles or composite nanoparticles [150,151,152,153,154,155,156,157,158]. Additionally, carbon paste enzyme electrodes that were rich with oxygen or biocathodes diffused with air were employed to reduce errors occurring due to oxygen deficit [150,151,159,160,161,162].

The second generation of enzymatic electrochemical sensors relied on the utilization of an artificial mediator to overcome the oxygen deficit problems of the sensors preceding them. This included the use of either immobilization of glucose oxidase in a membrane, produced solution-based mediators, or redox-conducting polymers that can transfer electrons to and from the active site. Sensors developed in this regard utilize conductive organic salts, carbon nanotubes, binding proteins, and stabilizing artificial mediators [46,138,163,164,165,166,167,168,169,170,171,172,173,174,175,176].

The third generation includes recent advancements in the domain of electrochemical biosensors and utilizes the concept of direct energy transfer or DET. The major problem that needs to be rectified is minimizing the electron transport pathway between the oxidation-reduction site and the electrode surface [177,178,179,180,181]. The main strategies employed so far include reconstructing the apo-proteins on enzymes modified with co-factors, reconstructing the apo-enzymes on gold (Au) nanoparticles [178,179,182,183,184], attachment of enzyme active sites covalently to electrode surface 170, transformation of oxidase enzyme to hydrogenase by implantation of Platinum nanoparticles [185], thermodynamic reduction in Pt salts to Pt nanoclusters for the purpose of electrically wiring enzyme to electrode [185], covalent immobilization of glucose oxidase onto carbon nanotubes [186], production of amine functionalized carbon fiber nanotube for adsorption of glucose oxidase [187], site-specific modification of glucose oxidase [188], glucose oxidase immobilization using AuNP supported on functionalized nano-silica surface [189], production of an electrode array incorporated with carbon quantum dots [190] and utilization of graphene and multiwalled carbon nanotubes along with immobilization of glucose oxidase in Nafion [191].

Organic transistors produced via printable techniques also gain traction. One significant example is the production of printable organic transistor-based glucose sensing modes addressing design prospects and strategies in the glucose sensing field. Another is the development of a rapid dispense printed flexible interdigitated electrode. The electrode is modified with rGO-TiO2 nanohybrid for efficient signal processing and glucose detection [192,193].

A notable concern in the case of electrochemical sensors is the modeling of the required sensors. Computational modeling techniques, such as the use of CAD tools, machine learning, simulations, etc., prove to be exceptionally useful in addressing this concern. A notable example in this regard is the 2D modeling of a thin film transistor for glucose sensing, simulated using COMSOL Multiphysics version 6.2 [194].

#### 3.4.5. Electric/Electromagnetic Methods

Electric/Electromagnetic methods utilize various electromagnetic waves to probe biological tissues and extract information related to glucose concentration. Concepts and techniques used include ultrasound, impedance, spectroscopy, and waves (both micro and millimeter).

NIR and MIR absorption spectroscopy combines near-infrared and mid-infrared technology along with machine learning to analyze glucose absorption in tissues [82,87,89]. Time of flight and thermal emissions spectroscopy essentially determine times taken for light to travel when interacting with glucose as well as radiation emitted by the body when illuminated with light at a wavelength of 9.4 µm. This evaluates blood glucose levels due to changes in heat distribution [70,195].

Dielectric or impedance spectroscopy utilizes polarity and dielectric properties of glucose within the microwave range but analyzed at various frequencies to observe and correlate glucose levels [196,197,198,199]. Another electromagnetic approach is the use of radio frequency (RF) spectroscopy in which interdigital transducers or IDTs as well as stepped impedance resonators or SIRs are used to analyze electromagnetic wave interactions with tissues to analyze glucose concentrations [92,196,198,200,201,202,203]. Bio-impedance spectroscopy involves the application of alternating current and then measuring resultant conductivity [204,205]. Acoustic impedance evaluates the propagation time of ultrasound waves which are affected when there is an increase in concentration of glucose. This is essentially an ultrasound-based technique for glucose level measurement [206,207].

Transimpedance has started serving as a mechanism for the amplification and effective transmission of extracted data. Such mechanisms provide embedded signal conditioning circuits for noise-resistant glucose data transmission [208].

Millimeter waves and microwave-based glucose detection technology are based on the vibrational and radar or “bounce back” properties of glucose when light of different wavelengths is emitted on it. W-band spectrometer and diffusion-limited aggregation techniques are to be applied for effective management [201,209,210,211,212,213,214,215,216]. An example is the development of sensor utilizing a Complementary Split-Ring Resonator (CSRR) integrated with an inter-digital capacitor for glucose sensing.

Though the electromagnetic sensors hold promising results, the performance of the sensors (selectivity, sensitivity, and accuracy) and their user-friendliness still need to be improved. Advancements in wireless technology may lead to and enable less data transmission. Additionally, vector network analyzers need to be eliminated to provide compactness and portability.

#### 3.4.6. Nano Technology

**Figure 10:**
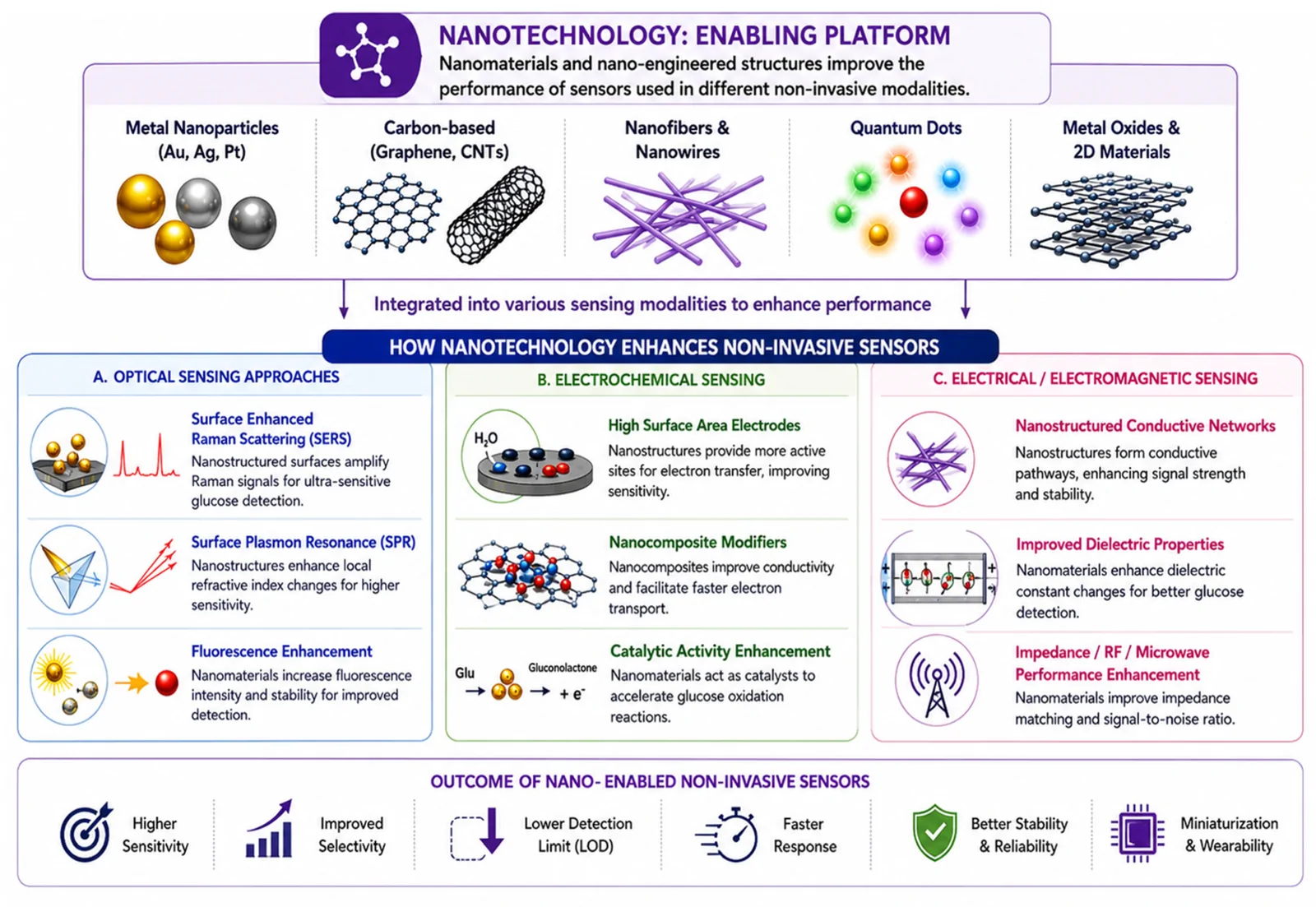
A compilation of nanotechnology and textile-based approaches being employed for non-invasive monitoring of glucose.

Figure 10 shows how nanotechnology can be used as an enabling platform for enhancing the performance of non-invasive glucose sensing systems. The framework has three parts: (1) nanomaterial platforms, (2) incorporation of sensing mechanisms and (3) effects on the performance of the sensor.

The first one is a selection of the major classes of nanomaterials including carbon-based nanostructures (graphene and carbon nanotubes), metallic nanoparticles (gold, silver and platinum), nanofibers and nanostructures, quantum dots and new two-dimensional materials. The nanomaterials have special physicochemical properties such as high surface area, high conductivity, tunable optical properties, and catalytic activity.

The second one illustrates the integration of these nanomaterials into various sensing mechanisms. In optical sensing, nanomaterials are utilized to improve the generation of the optical signal using various approaches, including SERS, SPR, fluorescence enhancement and optical signal amplification. Nanomaterials enhance the electron transfer and catalytic activity, active surface area and detection sensitivity in electrochemical sensors. They also improve the transduction mechanism via impedance modulation, conductivity, piezoelectric responses and mechanical flexibility.

The third component focuses on the benefits that might emerge from the integration of nanostructures, such as increased sensitivity and selectivity, improved signal-to-noise ratio, lower detection limits, faster response times, greater stability and reproducibility, miniaturization, wearable integration and greater accuracy in complex biological environments. Hence, nanotechnology is not a glucose sensing modality but a platform or a supporting technology for the performance of glucose sensing.

The nanoplasmonic techniques utilize a combination of optical concepts and nanotechnology to produce sensors that are readily wearable. This includes surface-enhanced Raman scattering (SERS), surface plasmon resonance (SPR), and plasmon-enhanced fluorescence as explained in optical methods.

Various nanoparticles have been employed for the development of smart wearables that can be worn for longer periods for continuous glucose monitoring. Carbon-based nanoparticles use carbon quantum dots (CQD), which are essentially a type of zero-dimensional nanomaterials with less than 10 nm in size and possess impressive fluorescence properties [217]. The CQDs provide differences in glucose concentration via spectral shifts and intensity alterations occurring due to different glucose concentrations. Other carbon-based approaches involve carbon nanotubes (single wall or double wall) as well as carbon allotropes such as graphene or fullerene. These structures are made more efficient when sensitivity and glucose selectivity are increased by the deposition and/or utilization of nickel oxide (NiO), platinum (Pt), palladium (Pd), alizarin, amino phenylboronic acid (ARS-PBA), chitosan, tin sulfide, reduced graphene oxide, indium tin oxide, and molybdenum disulfide [218,219,220,221,222,223,224,225].

Nanoparticles being utilized for the measurement of glucose other than carbon include nickel, cobalt, copper, and noble metal-based materials. The electron transfer during the electrooxidation process of glucose and the catalysis effect that nickel can perform during direct oxidation of glucose in alkaline conditions make it a significant transition metal in the non-invasive glucose monitoring technologies domain. Morchella-shaped Nickel-copper-layered double hydroxide hierarchal structures for non-enzymatic glucose sensing, Nickel foam (NF), and Nickel metal–organic framework (Ni-MOF) have been utilized so far for the measurement of glucose non-invasively. Nickel and its hierarchal structures in the form of nanosheets, nanowire arrays, and nano strands hold promising potential in the future for non-invasive glucose monitoring [226,227,228,229,230,231,232,233,234,235,236,237,238,239,240,241].

The nanostructure of cobalt in the form of nanoflowers utilizes cobalt alloy, cobalt (I, III) oxide, cobalt (II) hydroxide, and cobalt oxyhydroxide for glucose sensing. The nanoflowers can be loaded onto carbon cloth and graphene, transition metal nitride, and phosphide materials are utilized for enhancing conductivity [242,243,244,245,246,247,248].

Copper oxide (CuO) nanostructures are highly sensitive, highly selective, and rapidly responding. Sensors developed in this regard include the integration of Zinc oxide (ZnO) nanorods, CuO nanospheres, Nafion, carbon, silver nanoparticles, and CuO-MOF (Copper oxide, metal–organic framework) for accurate and reliable measurement [249,250,251,252,253,254,255,256,257,258,259,260,261]. Additionally, noble metals-based non-enzymatic nano architectures include the use of palladium (palladium nanoparticles on ionic fibrillated meso-cage carbon) and silver (particularly Ag@ZIF-67) for the development of novel glucose sensors that demonstrate good stability with low toxicity and significant electrocatalytic activity [262,263,264,265,266].

Piezoelectric sensors are based on the principle of sensing changes in resonance frequency that occur due to interactions between the oscillating crystal and a biomolecule. For the fabrication process, polymers such as polylactic acid and polyvinylidene fluoride, etc., or the anisotropic crystals of zinc oxide (ZnO), aluminum nitride quartz, etc., may be used. Certain piezoelectric sensors may use antigens or antibodies for biorecognition as well. Aspergillus Niger spores are also being utilized as biocatalysts [267]. The sensor produced is a fully flexible wearable glucose sensor integrating biocompatible nanocellulose for effective glucose level determination and essentially consists of a biofuel cell and a flexible electronic device integrated with microbial nanocellulose [267,268,269,270,271].

The most tested physiological fluid so far for non-invasive sensing of glucose is sweat; therefore, it is no wonder that many of the glucose sensors developed via piezoelectric materials also utilize the same fluid. Work done includes colorimetric and electrochemical-based biosensors using piezoelectric materials and a hydrogel-based sensor with integrated piezoelectric material [106,268,269,270,272,273]. A closed-loop brain-machine interfaced biosensor using enzyme/zinc oxide has also been developed which requires no external power source and promises compatible results [274]. Another example of a self-powered glucose detection system utilizes the coupling effect of glucose oxidase/zinc oxide, produced because of the reaction between piezoelectric material and enzyme [275].

Non-invasive and minimally invasive glucose monitoring uses optical, acoustic, electrical, and electromagnetic methods. Optical techniques (NIRS, MIRS, OCT, PPG, Raman, Fluorescence) detect glucose via light absorption, scattering, or molecular vibrations, offering high sensitivity and wearable compatibility but their performance is affected by skin properties and motion shown in Table 5. Acoustic, ultrasound, and bio-impedance methods monitor tissue or fluid changes, while EM methods detect dielectric variations, providing real-time but calibration-sensitive measurements.

Several commercially available non-invasive glucose monitors (Table 6) demonstrate diverse sensing technologies. GlucoTrack (ultrasonic, electromagnetic, thermal) and SugarBEAT (reverse iontophoresis patch) are available in limited or selected markets. DiaMonTech D-Base uses mid-infrared photothermal spectroscopy and is approved for clinical use. Older devices like GlucoWatch Biographer (reverse iontophoresis) and Pendra (impedance spectroscopy) were briefly commercialized but later withdrawn. These examples highlight the range of technologies applied to real-world non-invasive glucose monitoring and the challenges of adoption.

### 3.5. Critical Analysis

The preceding pages explain the different methods, techniques and technologies being used and researched for the non-invasive monitoring of glucose, from a textile perspective. But what exactly are the merits of integrating the techniques into textiles and wearables is discussed in this section. For this purpose, textile integration with the non-invasive glucose monitoring techniques is contrasted with non-textile-based non-invasive glucose monitoring. Table 7 essentially serves as a list of pros and cons for the two integration approaches, and explains the opportunities and challenges presented by both. Analyzing and working on these challenges can lead to efficient advancements in the field of non-invasive glucose monitoring, both from a textile as well as a non-textile perspective.

By analyzing the potential of the two in different categories, aspects and factors and working to improve them in the aspects in which they lack, significant advancements can be made in the field of non-invasive glucose monitoring.

Now that the textile and non-textile-based non-invasive glucose monitoring perspectives have been compared, it seems only appropriate to provide a critical analysis of the different methods in the textile-based non-invasive field as explained in the paper. Table 8 summarizes representative technologies developed for non-invasive and wearable biosensing, with emphasis on optical, electrochemical, piezoelectric, and RF-based sensing approaches. The reported studies demonstrate the use of different transduction mechanisms for monitoring glucose and other physiologically relevant biomarkers, including sweat-based and blood-glucose sensing. The technologies exhibit diverse performance characteristics in terms of detection range, sensitivity, limit of detection, response time, and prediction accuracy. However, several challenges remain, including interference from complex biological environments, dependence on material composition and fabrication conditions, mechanical and long-term stability, the influence of physiological and anatomical variations, and limited validation in real human subjects. These limitations highlight the need for robust, selective, flexible, and clinically validated sensing platforms for reliable continuous health monitoring.

## 4. Conclusions

This review provides a comprehensive evaluation of glucose monitoring technologies for diabetic patients. This discussion was focused on non-invasive glucose monitoring technologies with some basic discussion on invasive and minimally invasive glucose monitoring technologies. A structured literature review methodology was employed, analyzing selected 325 peer-reviewed studies from 2018 to 2026 across optical, electrochemical, electromagnetic, nanotechnology-enabled, and hybrid sensing approaches. Articles were screened for relevance, quantitative performance metrics, sensor configurations, and physiological fluid utilization, and subsequently categorized for detailed comparison of textile-based and non-textile wearable systems.

The review highlights the strengths and limitations of various approaches, including optical methods (NIRS, MIRS, OCT, PPG, Raman, fluorescence), electrochemical sensors, nanotechnology-based devices, and electromagnetic techniques. Non-invasive methods prioritize patient comfort, continuous monitoring, and minimal physiological disruption, while minimally invasive approaches reduce the frequency and discomfort of blood sampling through microneedles, lenses, antennas, and surface plasmon resonance. Physiological fluids such as interstitial fluid, sweat, saliva, ocular fluid, and breath serve as alternative matrices, each with variable correlation with blood glucose, necessitating calibration, multi-modal signal integration, and machine learning algorithms to improve accuracy.

Textile-based systems enhance comfort, flexibility, and discretion, while non-textile devices such as wristbands and patches provide robust signal delivery and easier power integration. Despite significant advances, challenges remain in sensor durability, washability, calibration, selectivity, and long-term stability, which must be addressed for commercial viability. Current commercially available devices, including Abbott FreeStyle Freedom Lite, AgaMatrix WaveSense KeyNote, Arkray Glucocard X-meter, Bayer Ascensia Contour, Bionime Rightest GM-300, Nova Max, Roche Accu-Chek Aviva, and Advocate Redi-Code, demonstrate the practical translation of enzymatic and electrochemical principles with rapid test times.

Future research should focus on multi-modal wearable integration, combining optical, electrochemical, and nanotechnology approaches, supported by machine learning-driven calibration, self-powered systems, and flexible designs suitable for daily use. Standardized validation across physiological fluids and incorporation of robust, miniaturized electronics will be essential to enhance reliability, accuracy, and clinical adoption. Addressing these challenges, guided by the insights from this structured review, will advance the development of next-generation, patient-centered, non-invasive glucose monitoring wearables, bridging the gap between laboratory innovation and practical clinical applications, ultimately improving diabetes management and patient quality of life.

## Figures and Tables

**Table 1:** Commercially available glucose biosensors [30].

Manufacturer	Brand	Assay Method	Test Time (s)
Abbott	FreeStyle Freedom Lite	GDH-PQQ	~5
AgaMatrix	WaveSense KeyNote	GOD	4
Arkray	Glucocard X-meter	GDH	5
Bayer	Ascensia Contour	GDH-FAD	5
Bionime	Rightest GM-300	GOD	8
Nova Biomedical	Nova Max	GOD	5
Roche	Accu-Chek Aviva	GDH-PQQ	5
Diabestic Supply of Suncoast	Advocate Redi-Code	GOD	7

**Table 2:** Compilation of techniques used in minimally invasive glucose monitoring.

Tool/Technique	Biomarker Extracted	Description	Pros & Cons	References
Surface plasmon resonance (SPR)	Interstitial fluid	Changes in glucose concentration levels can be determined by identifying small changes in refractive index in the interface which causes a shift in resonance frequency that leads to changes in intensity curve.	Pros: Highly sensitive to glucose levels. Cons: Sensitive to motion, sweat and temperature. It is harder to calibrate.	[18,47,48]
Optical polarimetry	Tears	Uses concept of chirality of glucose as it can rotate the polarization plane of a light beam.	Pros: High resolution. It can be easily miniaturized. Cons: Sensitive to temperature, motion and other optically active compounds.	[18,37,49,50]
Microneedle arrays	Interstitial fluids	Can use hydrogels, metals, silicones, polymer to extract Interstitial fluid and sense biomarkers such as glucose, lactate $\mathrm{ErbB}2^{2}$.	Pros: Continuous glucose monitoring, accuracy and efficiency. Cons: Fear of use and expense	[32,42,43,44]
Indwelling ISF sensors	Interstitial fluid	Uses implantable electromagnetic sensors, circuits, antennas, etc., to sense variance in interstitial fluid.	Pros: Accurate and continuous for upto 30 days. Cons: Fear of use and cost.	[32,39,40,51]
Sonophoresis	Interstitial fluid	Transdermal provision of drugs by ultrasound	Pros: Accuracy Cons: Not well developed yet.	[38,42]
Lens	Tears	Determination of glucose levels in ocular humor by the use of enzymatic anthropometric techniques	Pros: Miniaturized, continuous monitoring Cons: Psychological reluctance towards lens insertion, not much research and development done.	[37,38]

**Table 3:** Physiological fluids that can be utilized for glucose sensing via non-invasive methods.

Physiological Fluid	Typical Glucose/Biomarker Range	Units	Relationship to Blood Glucose	Major Challenges	Representative References
Sweat	Typically reported in the µM–mM range depending on sweat rate, sampling method, and physiological condition	µM–mM	Moderate correlation; influenced by sweat secretion dynamics	Variable sweat production, evaporation, contamination, pH effects	[52,53]
Saliva	Generally lower than blood glucose and highly dependent on salivary flow rate	µM–mM	Moderate to weak correlation	Food contamination, oral health status, dilution effects	[54]
Tear Fluid	Lower glucose concentration than blood but demonstrates measurable correlation	µM–mM	Moderate correlation; suitable for ocular sensing platforms	Limited sample volume, irritation, variability in tear secretion	[55,56]
Urine	Glucose appears primarily when renal threshold is exceeded	mM	Indirect indicator of hyperglycemia rather than real-time blood glucose	Delayed response, hydration effects, unsuitable for continuous monitoring	[57]
Breath (Acetone)	Typically reported as volatile acetone concentration rather than glucose concentration	ppm	Indirect metabolic marker associated with glucose metabolism and ketosis	Strongly influenced by diet, metabolism, and environmental conditions	[58]

**Table 4:** Role of microfluidic systems in wearable non-invasive glucose monitoring.

Function	Microfluidic Contribution	Benefit	References
Sweat Collection	Passive capillary channels and reservoirs	Continuous sample acquisition without external pumps	[59,60]
Sample Transport	Controlled movement through microchannels	Reduced contamination and evaporation	[59,61]
Sample Refreshing	One-way flow pathways and collection chambers	Prevention of mixing fresh and old samples	[60,61]
Multiplexed Monitoring	Multiple sensing chambers for glucose, pH, electrolytes and temperature	Improved analytical reliability and calibration	[63,64]
Saliva Sampling	Directed transport of low-volume saliva samples	Reduced contamination and improved sensor contact	[65]
Tear Fluid Collection	Integrated microfluidic contact lens systems	Enhanced tear–sensor interaction	[66]
Wearability	Flexible and stretchable microfluidic substrates	Improved comfort and long-term use	[59,62]

**Table 5:** Sensing tool/techniques and their pros and cons.

Tool/Technique	Biomarker/Sensing Principle	Description	Pros & Cons
Near-Infrared Spectroscopy (NIRS) [276]	Optical absorption of glucose in tissue	Uses light (750 nm–2.5 μm) transmitted through skin; glucose concentration is estimated from absorption spectra.	Pros: Painless, rapid, suitable for wearable devices. Cons: Sensitive to skin thickness, temperature, and motion artifacts.
Mid-Infrared Spectroscopy (MIRS) [277]	Glucose-specific absorption peaks	Measures glucose absorption in the mid-infrared region (2.5–25 μm) using optical lasers.	Pros: High glucose specificity. Cons: Limited penetration depth and expensive instrumentation.
Optical Coherence Tomography (OCT) [278]	Optical scattering and refractive index changes	Detects tissue structural changes caused by variations in glucose concentration.	Pros: High resolution and sensitivity. Cons: Complex signal processing and calibration required.
Optical Polarimetry [279,280]	Optical rotation of polarized light	Exploits the chiral nature of glucose to measure rotation of polarized light, particularly in ocular fluids.	Pros: Label-free and non-contact. Cons: Interference from other optically active substances.
Photoplethysmography (PPG) [281]	Blood volume and optical changes	Uses infrared or visible light to monitor glucose-related changes in blood flow characteristics.	Pros: Easily integrated into smartwatches and wearables. Cons: Lower specificity and susceptibility to motion artifacts.
Raman Spectroscopy/SERS [282]	Molecular vibrational signatures	Measures glucose-specific Raman scattering patterns; SERS enhances signal strength using nanostructures.	Pros: High molecular specificity. Cons: Weak native glucose signal and high equipment cost.
Photoacoustic Spectroscopy [283]	Acoustic waves generated by glucose absorption	Detects ultrasound signals generated when glucose absorbs pulsed light.	Pros: Improved penetration depth and sensitivity. Cons: Complex instrumentation.
Surface Plasmon Resonance (SPR) [284]	Refractive index variation	Detects glucose-induced changes in refractive index near a metallic sensing surface.	Pros: Highly sensitive and real-time monitoring. Cons: Sensitive to environmental variations and motion.
Fluorescence-Based Sensors [285]	Fluorescence intensity changes	Utilizes fluorescent nanoparticles or quantum dots whose emission changes with glucose concentration.	Pros: High sensitivity and miniaturization potential. Cons: Photobleaching and stability concerns.
Bio-Impedance Spectroscopy [286]	Electrical conductivity and dielectric properties	Applies alternating current and measures tissue impedance changes correlated with glucose levels.	Pros: Low power consumption and wearable compatibility. Cons: Influenced by hydration status and skin properties.
Microwave/Millimeter-Wave Sensing [287]	Dielectric property changes	Measures alterations in electromagnetic wave propagation caused by glucose concentration changes.	Pros: Continuous and non-contact monitoring possible. Cons: Accuracy and selectivity require further improvement.
Ultrasound/Acoustic Impedance [288]	Acoustic propagation characteristics	Detects glucose-related changes in ultrasound velocity and acoustic impedance of tissues.	Pros: Safe and non-invasive. Cons: Signal affected by tissue heterogeneity.

**Table 6:** Commercially available glucose biosensors for non-invasive monitoring.

Device	Company	Technology	Commercial Status	Reference
GlucoTrack	GlucoTrack Inc., Rutherford, NJ 07070, USA	Ultrasonic, Electromagnetic & Thermal sensing	Commercially available (limited markets)	[289]
SugarBEAT	Nemaura Medical Inc., Manhattan, New York, NY 10019, USA	Reverse iontophoresis skin patch	Commercialized in selected markets	[290]
DiaMonTech D-Base	DiaMonTech GmbH, 10245 Berlin, Germany	Mid-infrared photothermal spectroscopy	Commercial clinical device	[291]
GlucoWatch Biographer	Cygnus Inc., Redwood City, CA 94063, USA	Reverse iontophoresis	FDA-approved commercial device (discontinued)	[292],
Pendra	Pendragon Medical AG, CH-8050 Zürich, Switzerland	Impedance spectroscopy	Commercialized briefly, later withdrawn	[291]

**Table 7:** Comparison of textile and non-textile-based non-invasive glucose monitoring.

Factor/Aspect	Textile-Based Monitoring	Non-Textile-Based Monitoring
Description	Integration of non-invasive glucose monitoring techniques and technologies (optical fibers, conductive ink for electrochemical sensors) into textile medium to essentially create “smart clothing” or “electronic textile.” [293]	Integration of non-invasive glucose monitoring techniques and technologies into smart watches and ear canal devices, etc. [294]
Wearability & comfort	Flexibility and breathability enhance comfort & wearability. Discretion can be enhanced by integration into daily wear items not visible to others such as upper-arm bands, undershirts, etc. [295]	Rigid nature reduces wear comfort while bulky nature makes it easily apparent. [296]
Manufacturing	Good potential for scalable manufacturing, especially in the case of electrochemical methods since fabrication techniques such as stencils, screen-printing, weaving optical fibers into final product are compatible with the production of non-invasive wearables. Good potential for relatively low-cost manufacturing in the case of electrochemical-based sensors. Electromagnetic, nanotechnology and optical techniques utilize costly materials and thus, lead to higher manufacturing costs. [297]	Manufacturing is already being performed. Utilization of miniaturized, high-precision and complex materials leads to higher manufacturing costs. [298]
Sensing source	Most of the research targets sweat as sensing medium/source since it is readily available, can provide accurate results, does not require an invasive source to be inserted into the body for its extraction and is not damaging to textile substrates. [299]	Significant research has been performed utilizing sweat, breath, ocular fluid, and saliva, etc., for extraction of glucose levels. [300]
Power Integration	Can support low-power sensing Self-powering systems have also been research upon (as explained in Section 3.4.6). Can be integrated with multi-modal systems for data cross-validation benefits. Power supply and data transmission systems/modules need to be attached rigidly—compromising comfort and discretion perspective. [301]	Easier integration of power mediums such as batteries, data processing chips, etc. Robust power devices. [302]
Signal	Poor signal consistency. Poor signal delivering mechanisms. Highly susceptible to stretching, movement and textile-substrate distortion during use. [303]	Robust signal and data delivery mechanisms. Not susceptible to stretching, and movement. [304]
Durability	Easy degradation upon washing, friction, skin contact, and interaction with bodily fluids. Extremely short life span. [305,306]	Relatively better durability, life span and shelf life due to robust outer and inner structures. [307]
Response	Wearables have time lags since time is required for sufficient physiological fluid extraction required for a proper response. [308]	Medium/device using biofluids face significant time lag challenge. While devices employing optical or electromagnetic techniques are relatively better in this regard. [309]
Accuracy	Weak variable correlation to blood glucose levels, thus resulting in lower clinical accuracy. [310]	Better signal reliability since rigid structures ensure proper and consistent skin contact. Devices using interstitial fluid for glucose extraction have good accuracy comparable to traditional methods. [311,312]
Commercialization potential	Mass production can be performed but should be standardized. Batch testing must be performed if the products are to be sold commercially. Psychological barriers are to be reduced via effective marketing campaigns since population might consider it less accurate than blood-based glucose monitoring. [313]	Good potential since compatible accuracy to traditional methods Higher unit cost may pose a threat to wide-set commercialization. [314]
Development focus/challenges to address	Overcoming durability issues Improving accuracy Addressing time lag and signal inconsistency concerns Focus on self-powering systems Miniaturize data transmission modules Improve life span and shelf life [315]	Enhancing accuracy against physiological interferences. Improving unit cost by introducing improved, standardized manufacturing systems. Reducing bulkiness and conspicuity by focusing on miniaturization. Enhancement of wear comfort by introducing flexible systems. [316]

**Table 8:** Critical analysis of textile-based non-invasive glucose monitoring methods.

Technology	Description	Key Limitation
Near-Infrared Spectroscopy (NIRS) [317]	Non-invasive optical technique using 650–1000 nm light to measure tissue oxygenation through oxygenated/deoxygenated hemoglobin absorption. Reported rSO_2_ values of 50.5–86.0%; the sternum was the most reliable site (74.35 ± 2.96%, ICC = 0.669).	Results are affected by sensor placement, anatomical site, age, sex, tissue properties, and device type; findings were specific to the Masimo O3 NIRS sensor
Mid-Infrared Spectroscopy (MIRS) [318]	THz metamaterial optical sensor using MXene, black phosphorus, and graphene resonators for protein/brain tumor biomarker detection. Achieved 395 GHz/RIU sensitivity, FWHM = 0.037 THz, Q-factor = 8.649–9.108, and ML prediction accuracy of R^2^ ≈ 86% for refractive index and 96% for angular dependence.	Primarily a theoretical/numerical study; requires improved signal-to-noise ratio, clinical validation with real biomarkers, and surface functionalization.
Optical Polarimetry + Machine Learning [319]	Non-invasive optical method that measures glucose-related optical rotation from reflected light. The system uses multiple wavelengths and intensity levels, with machine learning for glucose prediction. Reported R^2^ = 86–100% for refractive-index prediction and ≈96% for angular dependence.	Optical signals are strongly affected by skin scattering, tissue properties, skin tone/thickness, and other biological substances, which can reduce accuracy and require robust ML models.
Ni-MOF Electrochemical Sensor [320]	Non-enzymatic sweat glucose sensor based on a longitudinally expanded Ni-MOF. Linear range: 0–0.5 mM glucose; detection was performed at 0.75 V using an all-solid-state sensor. Ultrasonication enhanced Ni(IV)-based catalytic oxidation of glucose.	Requires improved selectivity and anti-interference performance for complex sweat samples
Nickel Nanosheet Electrochemical Sensor [321]	Non-enzymatic wearable sweat ascorbic acid sensor based on Ni_3_N/Ni heterostructure supported on porous N-doped reduced graphene oxide (Ni_3_N/Ni/N-rGO). The sensor uses the high conductivity of N-rGO and enhanced interfacial electron transfer/catalytic activity of the Ni_3_N/Ni heterostructure. Detection range: 0.5–1937.5 μM; sensitivity: 663.87 μA mM^−1^ cm^−2^; LOD: 0.25 μM.	The sensor is highly dependent on the optimized nanocomposite composition and fabrication conditions (GO content, nitridation temperature, and calcination time), which may affect reproducibility and large-scale fabrication.
CuO Nanostructure Sensor [322]	Flexible conductometric sweat-loss sensor based on Al-doped CuO nanostructured thin films fabricated on cellulose acetate using the SILAR method. The optimized 2.0% Al-CuO sensor showed a sensing response of 3.35 toward 393 mM artificial sweat, LOD = 3.40 mM, and R^2^ = 0.985.	Sensor performance decreased by approximately 10% after 4 weeks and by ~13% under 40 mm bending, indicating limitations in long-term stability and mechanical durability.
Piezoelectric Hydrogel-Based Sensor [323]	Transparent PVA/BTCA/β-CD/GOx/AuNPs nanofiber hydrogel fabricated by electrospinning for wearable glucose monitoring. Linear range: 0.1–0.5 mM; sensitivity: 47.2 μA mM^−1^; LOD: 0.01 mM; response time: <15 s.	Limited linear detection range and dependence on enzymatic GOx activity, which may affect long-term stability.
Self-Powered Piezoelectric Sensor [324]	A GOx@ZnO nanowire-based piezo-enzymatic sensor that uses the piezoelectric output as both the glucose-sensing signal and the power source, enabling battery-free glucose monitoring. The sensor exhibited a linear glucose detection range of 0.024–0.119 g L^−1^ (0.13–0.66 mM) with a LOD of approximately 0.019 g L^−1^ (0.106 mM) and was demonstrated for in vivo blood-glucose monitoring in a mouse model.	The sensing performance can be affected by other electrolytes and metabolites in body fluids, while the system requires mechanical deformation for signal generation and remains limited by animal-level validation and long-term stability.
RF Spectroscopy Sensor [325]	Non-invasive glucose monitoring through resonance-frequency shifts caused by changes in dielectric properties; tested over 0–240 mg/dL glucose concentration with a LOD of 29.89 mg/dL and sensitivity of 264.2 kHz/(mg/dL).	Validated using a dual-layer PDMS microfluidic vascular phantom rather than human in vivo testing; cable positioning and physiological factors may affect accuracy.

## Data Availability

No new datasets were generated or analyzed during the current study. All data discussed in this review are available in the cited published literature.
